# Associations between capacity of cognitive control and sleep quality: a two-wave longitudinal study

**DOI:** 10.3389/fpsyg.2024.1391761

**Published:** 2024-06-17

**Authors:** Yongchun Wang, Huanping Lin, Xiqin Liu, Bojia Zhu, Meihui He, Caiqi Chen

**Affiliations:** ^1^School of Psychology, South China Normal University, Guangzhou, China; ^2^Key Laboratory of Brain, Cognition and Education Sciences, Ministry of Education, South China Normal University, Guangzhou, China; ^3^Center for Studies of Psychological Application, South China Normal University, Guangzhou, China; ^4^Guangdong Key Laboratory of Mental Health and Cognitive Science, South China Normal University, Guangzhou, China; ^5^School of Foreign Languages, South China University of Technology, Guangzhou, China; ^6^Department of Human Resource, Guangzhou Branch of China Mobile Group Guangdong Company Limited, Guangzhou, China

**Keywords:** capacity of cognitive control, trait mindfulness, emotional distress, sleep quality, sequential mediation

## Abstract

This longitudinal study explored the impact of the upper limit of cognitive control on the sleep quality of high school students. We collected data in two waves to examine four main variables: capacity of cognitive control (CCC), trait mindfulness, emotional distress and sleep quality. At the first time point (T1), trait mindfulness and emotional distress were measured by rating scales, and the CCC was evaluated by revised backward masking majority function task. Sleep quality was rated 5 months later (T2). The results indicated that: (1) the CCC was negatively correlated with trait mindfulness, and trait mindfulness was negatively correlated with emotional stress; (2) there was no simple mediation of either trait mindfulness or emotional distress in the relationship between CCC and sleep quality; (3) instead, the CCC was associated with poor sleep quality in a sequential mediation through trait mindfulness and then emotional stress. The research highlights the importance of trait mindfulness and emotional distress for addressing sleep problems in adolescents.

## 1 Introduction

Sleep is a vital physiological process that plays an indispensable role in the psychosocial adjustment of individuals (Sarchiapone et al., 2014). However, sleep problems are becoming a growing concern among adolescents (Gradisar et al., 2022). The chronic deprivation of sleep or its inadequate quality can have negative effects on the cognitive and emotional functioning of adolescents (Williams et al., 2013). Therefore, understanding the factors behind sleep problems is crucial for developing effective prevention and intervention strategies to improve individuals’ health and well-being in adolescence and beyond.

There is an abundance of research dedicated to identifying the factors that affect sleep quality. This research primarily focuses on three areas. First, numerous studies concentrate on the factors that impact sleep quality within specific demographics (e.g., Souders et al., 2009; Songkham et al., 2019; Bahani et al., 2024; Guller and Yaylaci, 2024). Secondly, while influences on sleep quality can originate from various aspects, a significant number of studies are centered around cognitive and emotional factors (e.g., Hsu et al., 2009; Burke et al., 2015; Mueller et al., 2024; Schantz et al., 2024; Xie et al., 2024). Last, applied research aimed at enhancing sleep quality also garners considerable interest among researchers (e.g., Yosiaki et al., 1998; Lai and Good, 2005; Liu et al., 2024; Luo et al., 2024).

The procrastination of bedtime due to the excessive use of electronic devices at night is one of the factors that may contribute to sleep problems (Liu et al., 2017; Vernon et al., 2018). Furthermore, the delayed duration of sleep due to bedtime procrastination has been attributed to a lack of self-control (Kroese et al., 2016). Individuals who experience difficulty in resisting temptation are often characterized by low self-control, which may lead to the tendency of bedtime procrastination (Punamäki et al., 2007; Kroese et al., 2016; Seo et al., 2017). This, in turn, can result in insufficient sleep and poor sleep quality (Kroese et al., 2016). All the above studies focused on the effects of procrastination on sleep, but procrastination is only a behavioral manifestation resulting from a failure of self-control, an underlying cause of sleep problems. Therefore, it is important to understand the underlying mechanisms that contribute to the link between self-control and sleep, in order to develop effective interventions aimed at promoting healthy sleep habits among individuals with low self-control.

By examining self-control as a trait rather than a state to identify its impact on sleep, Kroese et al. (2016) found self-regulation to be associated with insufficient sleep, and bedtime procrastination to act as a mediating variable. However, they measured the subjects’ self-regulation ability with a self-control scale, and such self-reports may not fully objectively reflect the self-control level. Furthermore, their research on self-regulation was restricted to the level of individual external behavior habits, and did not involve any higher level, such as the cognitive level. Therefore, our study attempts to explore the impact of cognitive control capacity on sleep quality. The purpose is to find any changes in the relationship between cognitive control capacity and sleep quality to understand its influencing factors better. This longitudial study aims to enrich the evidence for any changes in the relationship between adolescent cognitive control and sleep quality from a new perspective.

Cognitive control refers to the process of flexibly allocating mental resources to process important information according to the goal at hand (He et al., 2019). With conceptual overlap, cognitive control and self-control have a common process, and they are often measured using the same experiments (Goldstein and Volkow, 2011; Hofmann et al., 2012; Goschke and Bolte, 2014). Measuring cognitive control ability allows for an examination of the underlying factors influencing changes in sleep quality, and helps improve the ability to predict the impact of cognitive control on sleep quality (Fan, 2014; Wu et al., 2015, 2016; Mackie and Fan, 2016). The cognitive control capacity (CCC), which reflects an individual’s upper limit in terms of cognitive control ability, has been identified as a critical determinant of cognitive function (Fan, 2014; Wu et al., 2020). Based on the dual system model of self-control, individuals with high CCC may exhibit a more flexible and higher order of control in their decision-making and action (Hofmann et al., 2009). This control mechanism empowers individuals to overcome immediate stimulus control, thus enabling them to engage in more purposeful and goal-oriented behavior (Hofmann et al., 2009). The functionality of the self-control system is contingent upon control resources (Fazio and Towles-Schwen, 1999; Vohs, 2006; Evans, 2008). The limited resources theory of self-control (Baumeister et al., 1998) posits that cognitive control resources are finite. In the event that the availability of control resources becomes depleted, the self-control system may experience a collapse and subsequently malfunction (Hofmann et al., 2009). Therefore, it is reasonable to assume that individuals with high levels of self-control display a heightened sense of control in the domain of sleep. Such individuals are more apt to choose long-term rewards, particularly the enduring benefits of quality sleep, rather than immediate stimuli that could potentially interfere with their sleep, such as engaging in behaviors that disrupt sleep (Liu et al., 2014). Based on the above analysis, we propose the first hypothesis as below.

*H1*: The capacity of cognitive control was positively correlated with sleep quality.

Studies have demonstrated that interventions aimed at promoting mindfulness are capable of enhancing sleep quality (Eisenlohr-Moul et al., 2016; Conley et al., 2018). Trait mindfulness is a construct that is closely linked to mindfulness, as it can be viewed as a natural progression of mindfulness practice, which refers to an individual’s ability to remain aware of and focused on the present experience (Liu et al., 2018). It is perhaps the most relevant personality trait to date for meditation-based interventions, used in many fields such as medicine and psychological interventions (Zeidan et al., 2010; Aivaliotis et al., 2017; Vignaud et al., 2018). Literature shows that the degree of trait mindfulness may have a significant correlation with the quality of sleep, whereby a greater level of mindfulness is positively associated with improved sleep quality. This observation is supported by empirical evidence suggesting that mindfulness practices, when consistently practiced, can enhance the development of trait mindfulness, which can in turn confer a range of benefits, especially in the context of sleep difficulties (Galla, 2016; Brisbon and Lachman, 2017; Xiao et al., 2019).

The concept of trait mindfulness is intrinsically connected to cognitive control and contains several components that are essential to enhance this control. People with high cognitive control abilities may have some special characteristics, such as a higher level of trait mindfulness, due to the regulation of attention promoting non-refined awareness of thoughts, emotions, and sensations. The direct, non-judgmental awareness and experience of mental and physical events in the present moment constitute the essence of trait mindfulness (Teasdale et al., 1995). The awakening of mindfulness requires a cognitive control process, i.e., attention self-regulation (Bishop et al., 2004).

In addition, the strength model of self-regulation assumes that the ability to self-control depends on limited, domain-independent resources (Baumeister et al., 2007). According to the model, self-regulation is a limited resource. Like muscle strength, it needs to relax once it is exhausted. Any effort of self-control will temporarily reduce this resource, resulting in a state of exhaustion of self-regulation which makes self-control more likely to fail in any subsequent self-control attempt. When individuals engage in mindfulness activities, the non-judgmental attitude will cause individuals to consume self-control resources. When negative emotions arise and individuals choose not to judge them, it is like the experiment by Baumeister et al. (1998) in which individuals in a room with biscuits, if allowed to smell or eat carrots only, would reduce their strength of self-regulation. Furthermore, self-regulation, like muscle strength, can be improved through long-term exercise.

Based on the above analysis, the study proposes a second hypothesis.

*H2*: Trait mindfulness mediates the positive correlation between the capacity of cognitive control and sleep quality.

In addition, there is evidence that sleep quality is strongly associated with anxiety and depression (Gadie et al., 2017). Many clinical studies on adolescents report that reduced sleep duration may be associated with emotional problems such as depression and anxiety symptoms (Hall et al., 2000; Zhai et al., 2021). The quality of an individual’s sleep was found to be negatively associated with a negative mood before going to bed (Shen et al., 2018) and positively associated with a positive mood (Latif et al., 2019). Research suggests that negative emotions may hinder an individual’s ability to perceive the benefits of adhering to a regular sleep schedule, including increased energy levels and improved mental health. Specifically, negative emotions could lead individuals to perceive the rewards associated with going to bed on time as distant and ineffective in helping them cope with their current negative emotional state (Sirois and Pychyl, 2013). As a prevalent phenomenon in the adolescent population, emotional distress is a commonly used indicator of mental health, which is often characterized by anxiety, depression, and somatic symptoms (Drapeau et al., 2012). Cognitive control was found to be consistently associated with emotions such as depression and anxiety (Tice and Bratslavsky, 2000; Pychyl and Sirois, 2016; Ebneabbasi et al., 2021). Compared with positive emotions, negative emotions may trigger more intense emotional experiences and may lead to the failure of self-control (Heatherton and Wagner, 2011). Following the limited resource theory of self-control (Baumeister et al., 1998, 2018), we assume that adolescents may deplete their self-control resources in emotional distress and the regulation of their distress, leaving a shortage of cognitive control resources that would otherwise be used to counteract delayed bedtime, which in turn would affect sleep quality (Baumeister et al., 1998). Emotional distress may be an influential factor in the relationship between cognitive control and sleep quality.

Therefore, individuals with more negative moods may prefer to replace the low-reward task (going to bed on time) with a more enjoyable task, like an entertaining media activity, to regulate their current mood (Sirois, 2014), which in turn may bring about sleep quality problems (Kroese et al., 2016).

Therefore, it is arguable that cognitive control capacity may delay sleep time and affect sleep quality through emotional distress. We would like to propose the third hypothesis as follows.

*H3*: Emotional distress mediates the positive correlation between cognitive control capacity and sleep quality.

Many researchers reported the inverse correlation between mindfulness and psychological distress in children and adolescents (Waters, 2016). Specifically, trait mindfulness was found to be negatively associated with depression and anxiety in students in Grades 4 to 7 (Lawlor et al., 2014), with anxiety in the general secondary school population (Li, 2017), and with depression in the post-trauma adolescents (Xu et al., 2018). Individuals with high levels of trait mindfulness exhibited reduced cortisol responses in high-stress situations (Brown et al., 2012). Furthermore, these individuals were also found to display lower resting activity in the bilateral amygdala and reduced gray matter density in the right amygdala (Way et al., 2010; Brown et al., 2012). Conversely, these neural patterns were shown to be positively associated with stress (Hölzel et al., 2010). Multiple meta-analyses demonstrate that such mindfulness-based interventions were effective in reducing depression and anxiety (Hofmann et al., 2010; Khoury et al., 2015). Stronger trait mindfulness was related to less depression and anxiety (Kiken and Shook, 2012), less rumination (Brown and Ryan, 2003; Kiken and Shook, 2014), fewer depressive negative cognitions (Gilbert and Christopher, 2010), and greater ability to release negative thoughts (Frewen et al., 2008).

Besides, according to the ego depletion theory (Baumeister et al., 2007)， any self-control activity of an individual is likely to consume self-control energy or resources, such as controlling impulses, controlling cognition (e.g., attention and thinking), controlling emotions and feelings, and making behavioral decisions. Baumeister et al. (1998) elucidates that the success or failure of volitional activities is affected by the amount of such resources. The more resources, the easier it is to execute successfully; the resources required for different volitional activities are the same. A series of seemingly different and unrelated activities may share the same resource. If resources are consumed in one volitional activity, then the actual resources available for another volitional activity will be reduced (Baumeister et al., 1998). In other words, in this study, the individual’s CCC level can be regarded as a stable control resource possessed by the individual. Individuals with high CCC levels can effectively control impulses (reduce unconscious attention) and have lower trait mindfulness levels (less conscious control of irrelevant stimuli). Mindfulness activities and controlling emotions will consume control resources. If the resources used by mindfulness activities are reduced, the resources available for controlling emotions will increase. Individuals’ effective control of emotional distress can lead to better sleep quality.

Therefore, this paper proposes a fourth hypothesis as follows.

*H4*: Trait mindfulness was positively correlated with emotional distress. They play sequential mediating roles in the positive correlation between the capacity of cognitive control and sleep quality.

**Figure 1:**
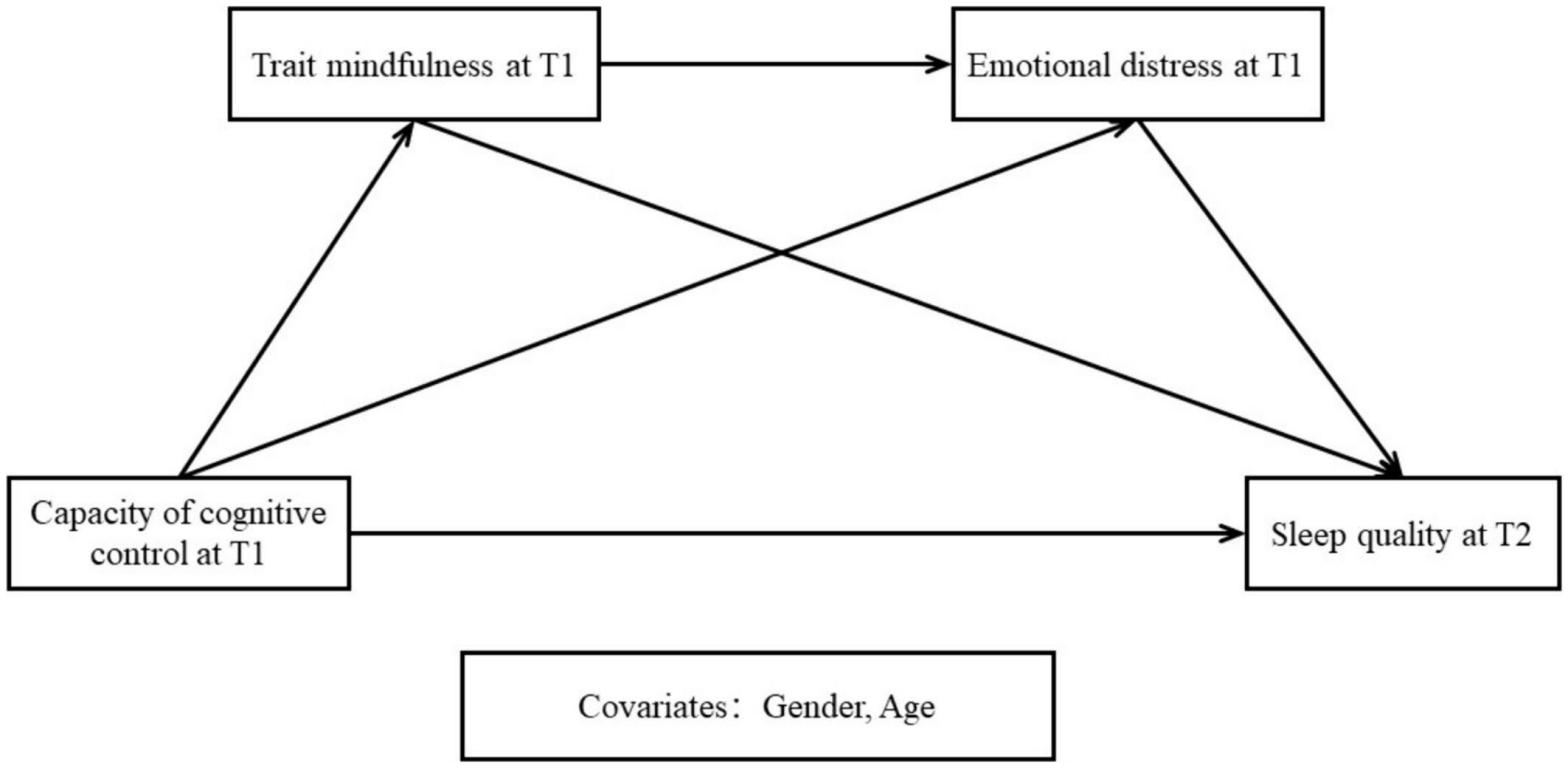
The effect of cognitive control capacity on sleep quality: A multiple-mediation model. T1: the first time point of data collection. T2: the second time point of data collection, conducted 5 months after the T1.

We attempt to understand the changes, if any, in the relationship between CCC and sleep quality, as well as its influencing factors. The mediating effects of both emotional distress and trait mindfulness are examined, which offers a new perspective to explore changes in the relationship between cognitive control and sleep quality in adolescents. In brief, a multiple mediation model (Figure 1) was conceptualized based on the above four hypotheses.

## 2 Methods

### 2.1 Participants

This study collected data in two rounds from students in Grade 10 from a high school in Guangdong Province in China. A total of 170 students participated in the initial round of data collection in September 2021 (T1). Five variables of interest were measured: CCC, trait mindfulness, emotional distress, gender and age. Questionnaire and experiment data were collected by professionally trained postgraduates and teachers in psychology. We first excluded 5 participants who wrongly responded to any of the three lie detection questions that were randomly assigned in questionnaires (i.e., “I make answers to these questions seriously.,” “I have never told a lie.,” “I can run a kilometer in 1 min”). Then we excluded two students with a diagnosis of psychiatry disorder (e.g., anxiety, depression) reported and another two students who did not successfully complete the MFT-M-R experiment. The final sample included 147 participants (48.99% female, *n* = 73). The sleep quality of all these participants was evaluated based on the second round of data collection in January 2022 (T2). Ethical approval was obtained from the Ethics Committee of the School of Psychology, South China Normal University. Written informed consent was provided by all participants. The researchers clarified the purpose of our research to the participants and assured them of the confidentiality and voluntary nature of the study. The tests and questionnaires were administered in the classroom during class time.

### 2.2 Measurements

#### 2.2.1 The revised backward masking majority function task

**Figure 2:**
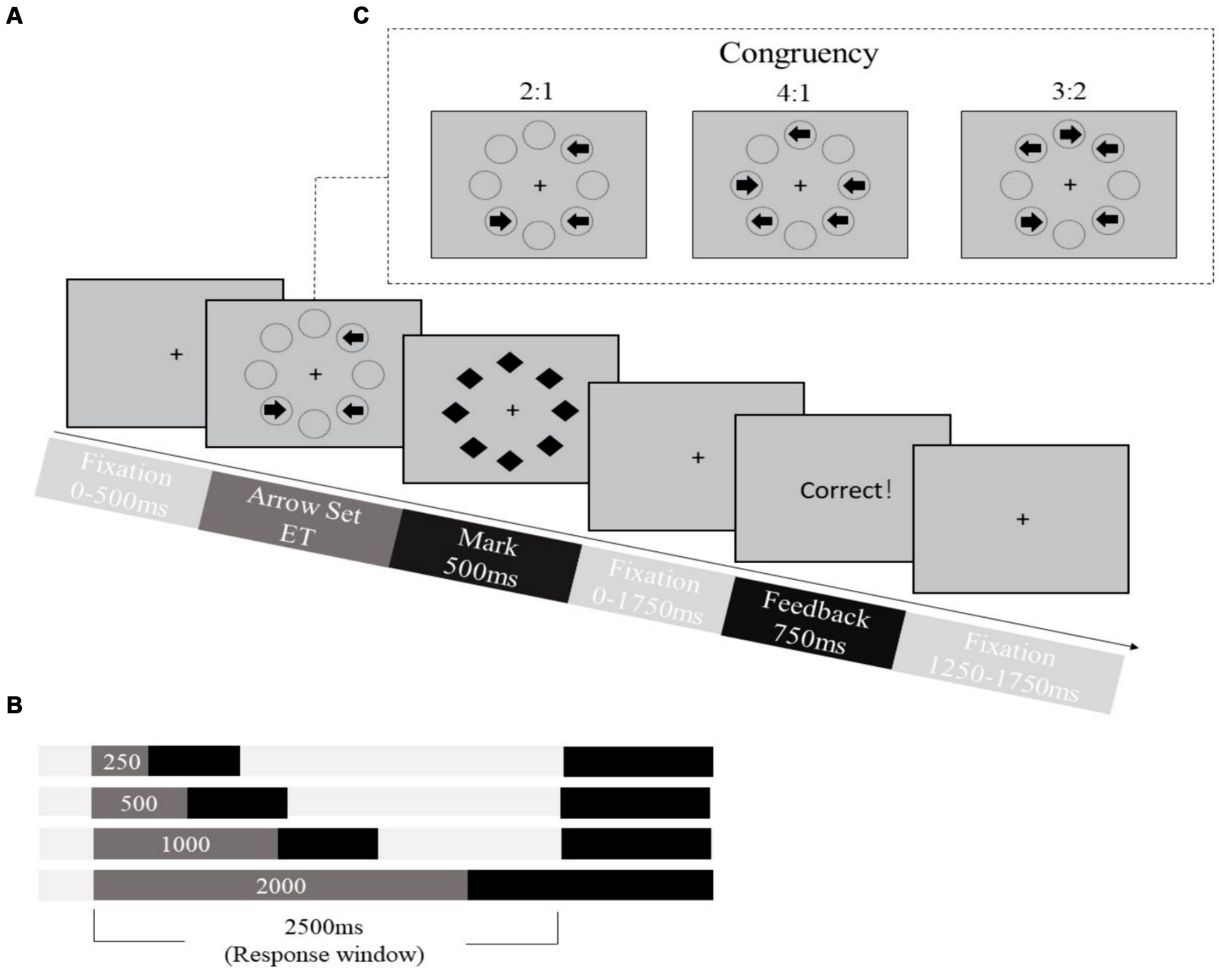
Stimuli and procedure of the backward masking majority function task (MFT-M). **(A)** Timeline of stimuli presentation in each trial (example of a trial with set ratio = 2:1). **(B)** Design of the exposure time (ET) of the arrow sets (pink bar) and the corresponding timeline. **(C)** Design of arrow sets with different ratios between the number of arrows pointing in the majority direction: the number of arrows pointing in the minority direction.

We used the revised backward masking majority function task (MFT-M-R) (Wu et al., 2016; He et al., 2022) to estimate the CCC of each participant. The stimuli and procedure of the MFT-M-R are shown in Figure 2. At the beginning of each trial, a central fixation was present for 0–500 milliseconds (ms), and then a set of left and right-pointing arrows appeared in eight possible locations around the fixation. The exposure time (ET) of these arrows was 250, 500, 1,000 or 2,000 ms, and the trial ended with a mask consisting of eight diamond shapes displayed for 500 ms at the same eight locations. After the masking disappears, a fixation of 0–1750 ms appears. Students were required to press a key to indicate the direction of most arrows pointing (“F” for left-pointing and “J” for right-pointing) as accurately and rapidly as possible, within a 2,500-ms window starting as the onset of the arrow set. If they could not identify the majority of arrow directions within the ET, they were instructed to guess the answer as a response. After the response window, 750-ms feedback would be given on the screen to tell whether the response was correct, followed by a post-stimulus fixation period of 1,000–1,500 ms. The total time of each trial was 6,300 ms. The length of the arrow and the diameter of each diamond was 0.37^°^ of visual angle while the radius from the fixation cross to the center of an arrow subtended approximately 1.5° of visual angle. The MFT-M-R was in a 3 (ET: 0.25, 0.5, 1, 2 s) × 6 (set ratio: 2:1, 4:1, 3:2) factorial design. The set ratio refers to the ratio between the number of arrows pointing to the majority direction versus the number of arrows pointing to the minority direction. The set size (total number of arrows) could be 3 or 5, and therefore the set ratio was 2:1 for the three-arrow set and 4:1, or 3:2 for the five-arrow set. This task is an adaptive test that terminates when the predetermined management length or measurement accuracy level is reached. Combining these two rules in MFT-M-R, 216 trials were used as the predetermined length, and SE was used as an indicator of measurement accuracy (SEs < 0.01–0.1 with intervals of 0.01). The SE in this study was 0.01. Participants could have a break between blocks. The entire task lasts about 20 min.

MFT-M-R was programmed to run on E-Prime (Version 1.3, Psychology Software Tools Inc., 2002; RRID: SCR_009567) and presented on a computer. Each participant was accompanied by an experimental assistant throughout the experiment to make sure that the task requirements were well understood. The assistant observed and recorded the participant’s behaviors.

#### 2.2.2 Sleep quality

This study used the Pittsburgh Sleep Quality Index (PSQI) to test the sleep quality of the sample. The scale has been developed and revised successively and has been proven to have good reliability and validity (*α* = 0.84) (Buysse et al., 1989; Liu et al., 1996). The PSQI consists of 19 self-evaluation items, 18 of which can be combined into 7 components, and the 19th item is not scored. Each component is scored from 0 to 3, and the cumulative score for each component is the total PSQI score (0 to 21 points). The evaluation period is the latest month, and the higher the score, the worse the sleep quality. The Cronbach’s α coefficient of this scale was 0.86, indicating excellent internal consistency.

#### 2.2.3 Emotional distress

A Chinese version of the Depression-Anxiety-Stress Scale or DASS-21 (Gong et al., 2010; Evans et al., 2020) was employed to collect information on emotional distress in the sample. The DASS-21 uses a four-point scale ranging from 0 (“does not apply to me at all”) to 3 (“applies to me most or most of the time”). The questionnaire includes three subscales (depression, anxiety and stress), each of which has seven items. All the 21 items on the DASS-21 add up to provide a measure of the overall emotional distress (Evans et al., 2020), ranging from 0 to 63. A higher score indicates a higher level of emotional distress. The Cronbach’s α coefficient of this scale was 0.94, indicating excellent internal consistency.

#### 2.2.4 Trait mindfulness

This study selected the five-facet mindfulness questionnaire (FFMQ) to measure the participants’ mindfulness level (Baer et al., 2008). Previous studies have shown that the scale has good reliability and validity in the Chinese middle school student population. The scale includes 39 questions, 20 of which are scored positively and 19 are scored negatively. FFMQ includes five factors: observing, describing, acting with awareness, nonjudging of inner experience, and nonreactivity to inner experience. The number of questions, respectively, included is 8, 8, 8, 8, and 7. The scale uses a 5-level rating. The mindfulness level is assessed by the total score of five dimensions, and the higher the total score, the higher the mindfulness level. The Cronbach’s α coefficient of this scale was 0.79, indicating excellent internal consistency.

#### 2.2.5 Controlled variables

Participants’ gender (female marked as 0; male marked as 1) were included as covariates in the analysis of all models. In addition, this research collected medical history information to rule out the impact of diseases on sleep quality. The above data are self-reported by students.

### 2.3 Statistical analysis

#### 2.3.1 The revised backward masking majority function task

Response time and accuracy rate were also computed and analyzed using MATLAB R2016b of Mathworks^1^ and IBM SPSS 22.0^2^. Any trial with no response was considered an invalid trial and was excluded from RT analysis. For each condition, trials with RT beyond three SDs of the average RT were regarded as outliers and also excluded from further analysis of RT. Each participant’s CCC was estimated based on the relationship between response accuracy and information rate (i.e., the amount of information needed to be processed in each second) (Wu et al., 2016). In brief, the amount of information conveyed by the arrow set was computed based on a perception decision-making strategy (grouping-search strategy), which is 2.58, 2.91, and 4.91 bit(s) for the 2:1, 4:1, and 3:2 ratio conditions, respectively. The information rate in each condition was computed as information amount divided by the ET, in the unit of bit per second (bps). The CCC was estimated as the information rate in which the accuracy started to drop, indicating the rate of information input began to exceed the capacity. Estimation of the CCC was implemented using a maximum likelihood estimation approach to fit the model of accuracy as a function of information amount and ET across all conditions, with CCC as the free parameter. The MATLAB script for estimating the CCC was downloaded from.

#### 2.3.2 Questionnaires

In all analyses, factors such as CCC, sleep quality, emotional distress, trait mindfulness, and age were treated as continuous variables, whereas gender was considered as a categorical variable (binary). We used SPSS 26 to examine descriptions and correlations between CCC, sleep quality, emotional distress, trait mindfulness, gender, and age. The data were standardized, and then mediation and moderation analyses were made using the process macro version 3.5 for SPSS (Hayes, 2017).

In the mediation model, CCC served as an independent variable (X), with subsequent sleep quality acting as the dependent variable (Y) while trait mindfulness (M1) and emotional distress (M2) as mediators. Covariates including gender and age were controlled in the analysis. To estimate the 95% bias-corrected confidence intervals (95% CI), a bootstrapping procedure with 10,000 iterations was performed. This approach allowed for a robust assessment of the mediation effects. To address potential common method bias, Harman’s One-Factor Test was conducted.

## 3 Results

### 3.1 Common method deviation test

Harman’s One-Factor Test was used to detect any possible common method bias. The results showed that the eigenvalues of 13 factors were greater than 1 and the factor with the largest eigenvalue explained 26.17%, which was less than 40%. Therefore, we believe that there was no significant common method bias.

### 3.2 Descriptive statistics and correlation analysis

Table 1 presents the results of descriptive statistics (means and standard deviations) and Pearson correlation analyses for the four main variables (CCC, PSQI, trait mindfulness and emotional distress) across the two time points. Specifically, CCC at T1 was negatively associated with trait mindfulness at T1 (*r* = −0.21, *p* < 0.001) and with gender at T1 (*r* = −0.18, *p* = 0.03). Similarly, trait mindfulness at T1 was negatively associated with emotional distress at T1 (*r* = −0.45, *p* < 0.001). However, there was a significant positive correlation between CCC at T1 and emotional distress at T1(*r* = 0.18, *p* = 0.02), and between emotional distress at T1 and poor sleep quality at T2 (*r* = 0.23, *p* = 0.01).

**Table 1:** Descriptive statistics and correlation analysis between variables.

	*M*	SD	Min	Max	1	2	3	4	5
1. CCC at T1	2.82	0.77	1.05	4.57	——				
2. Poor sleep quality at T2	5.39	3.67	0.00	20.00	−0.002				
3. Trait mindfulness at T1	3.14	0.55	1.67	4.67	−0.21**	−0.16			
4. Emotional distress at T1	14.46	11.80	0.00	63.00	0.18*	0.23**	−0.45**		
5. Gender	0.50	0.50	0.00	1.00	−0.16*	−0.07	0.10	−0.10	

*N* = 151. T1, Time 1; T2, Time 2. M, mean; SD, standard deviation. Age was expressed by years; Gender was coded with 0 for female and 1 for male. *p < 0.05; **p < 0.01; ***p < 0.001.

### 3.3 Mediation model test

**Figure 3:**
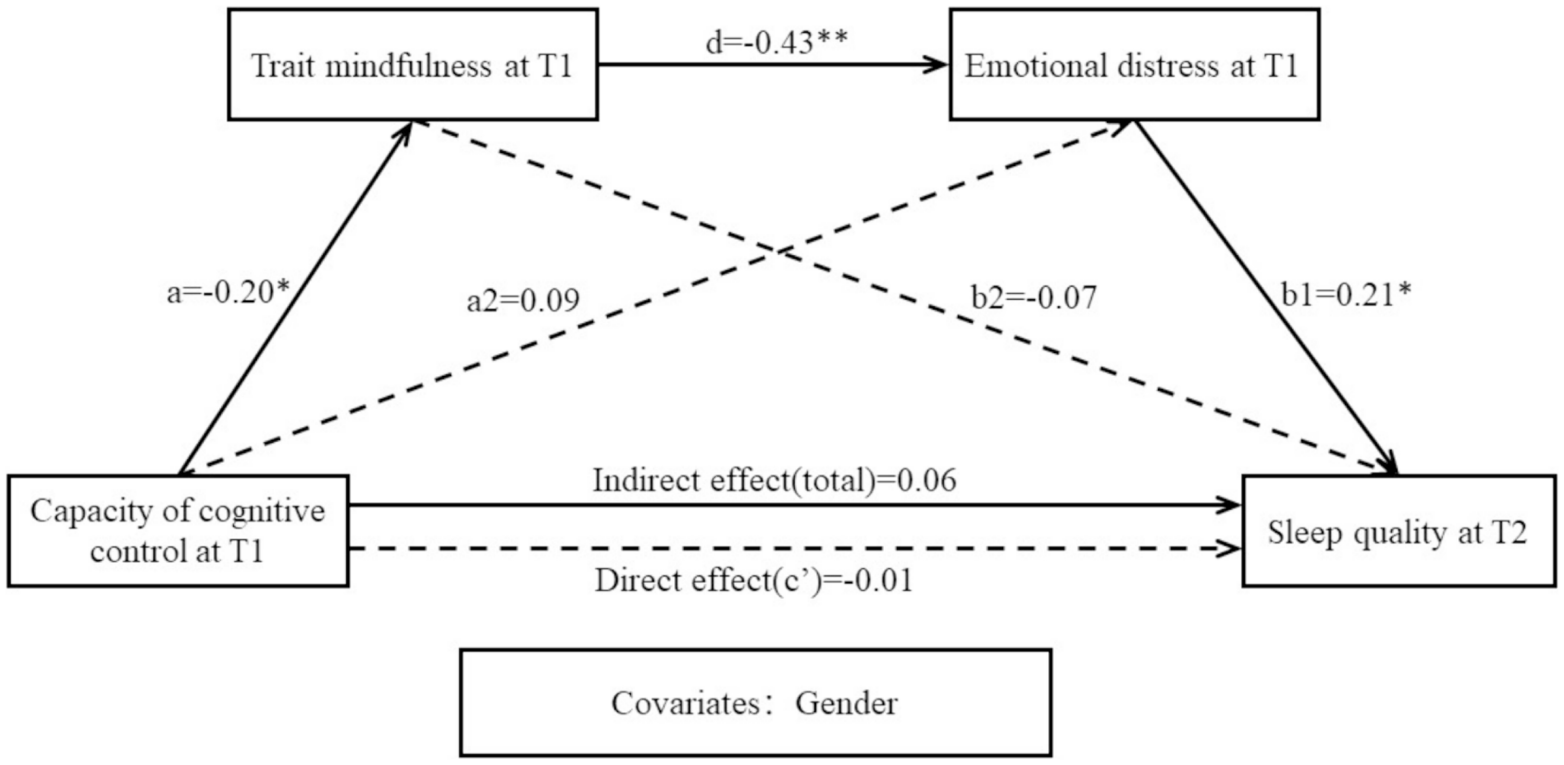
The mediation effects of trait mindfulness and emotional distress at T1 on the relationship between CCC at T1 and sleep quality at T2. *p < 0.05; **p < 0.01.

The results of the mediation analysis were showed in Table 2 and Figure 3, indicating that the association between CCC and poor sleep quality was mediated by trait mindfulness and emotional distress in sequence. The total effect model explained 0.47% of the variance in poor sleep quality at T2 (*R* = 0.07, MSE = 1.01, F2, 144 = 0.34, *p* = 0.71). The nonsignificant total effect suggests that CCC at T1 did not sufficiently explain poor sleep quality at T2 alone, so H1 was not supported. When considering trait mindfulness and emotional distress as mediators, path a1 showed a significant negative association between CCC and trait mindfulness at T1 (*β* = −0.20, SE = 0.08, *t* = −2.42, *p* = 0.02, 95% CI = [−0.37, −0.04]), with CCC accounting for 4.87% of the variance in trait mindfulness. However, since path b2 was not significant (*β* = −0.07, SE = 0.09, *t* = −0.74, *p* = 0.46, 95% CI = [−0.25, 0.11]), the indirect effect of trait mindfulness as a mediator was also nonsignificant, and H2 was rejected. Similarly, although path b1 revealed a significant positive correlation from emotional distress at T1 to poor sleep quality(PSQI) at T2 (*β* = 0.21, SE = 0.09, *t* = 2.29, *p* = 0.02, 95% CI = [0.03, 0.39]), path a2 was not significant (*β* = 0.09, SE = 0.08, *t* = 1.11, *p* = 0.27, 95% CI = [−0.07, 0.24]), so the indirect effect of emotional distress as a mediator was not significant. Therefore, H3 was not proved. However, path d was the same as path a1, which revealed a significant negative correlation from trait mindfulness at T1 to emotional distress at T1 (*β* = −0.43, SE = 0.08, *t* = −5.66, *p* < 0.001, 95% CI = [−0.58, 0.28]). The indirect effect of trait mindfulness and that of emotional distress as mediators were significant, so H4 was supported. Notably, the negative though nonsignificant direct effect (*β* = −0.10, SE = 0.08, *t* = −1.24, *p* = 0.22, 95% CI = [−0.27, 0.06]) along with the positive and significant total indirect effect (*β* = 0.06, SE = 0.03, 95% CI = [0.01, 0.13]) suggests a suppressing effect of emotional distress on the relationship between CCC at T1 and poor sleep quality at T2. The mediation model accounted for 7.44% of the variance in poor sleep quality at T2 (*R* = 0.25, MSE = 0.96, F4, 142 = 2.39, *p* = 0.05).

**Table 2:** Results of the multiple mediation analysis.

	Total effect model				Mediation model			
	***β***	**SE**	***t***	**95% CI**	***β***	**SE**	***t***	**95% CI**
CCC at T1	−0.13	0.08	−0.15	−0.18,0.15	−0.06	0.08	−0.74	−0.23, 0.10
Trait mindfulness at T1					−0.07	0.09	−0.74	−0.26, 0.11
Emotional distress at T1					0.21	0.09	2.29	0.03, 0.39
Gender	−0.07	0.08	−0.83	−0.24,0.10	−0.05	0.08	−0.62	−0.21, 0.11

All variables were measured at T1 except the dependent variable sleep quality. *p < 0.05; **p < 0.01.

## 4 Discussion

The present study constructed a sequential mediation model that demonstrated the roles of trait mindfulness and emotional distress in shaping poor sleep quality among high school students with high CCC. It may contribute to our understanding of how CCC impacts sleep quality. Our results revealed that CCC was indirectly associated with poor sleep quality in a sequential mediation first through trait mindfulness and then through emotional distress. It is worth noting that these studies were conducted on the assumption that the capacity of cognitive control was positively correlated with sleep quality. However, suppressing effects may exist in cases where the direct effect is not significant (MacKinnon et al., 2000). Because indirect and direct effects are indicated by different signs, one negative and the other positive,the direct effect of capacity of cognitive control on sleep quality was suppressed (Wen and Ye, 2014). Although the direct effect of CCC on later sleep quality was not significant, the indirect effect, due to the mediation of trait mindfulness and emotional distress effect, turned out to be significant. The most significant contribution of the current research may lie in its clarification of the sequential and indirect role of trait mindfulness and emotional distress as a mechanism explaining the link between CCC and sleep quality. However, neither trait mindfulness nor emotional distress independently mediated the relationship between CCC and sleep quality. This finding substantiates the strength model of self-control, which postulates that self-control relies on a finite energy reserve, and its exertion gradually depletes this reserve. This mechanism would make people with high CCC levels have fewer energy resources to allocate to other mental activities.

The present study extended previous research on CCC and sleep by showing that CCC was negatively (rather than positively) correlated with trait mindfulness. This result may be explained from the following two perspectives. On the one hand, the non-judgmental attitude is an important criterion for measuring the trait mindfulness, and it is related to automatic attention (Kabat-Zinn, 1990; Kabat-Zinn, 1994). Studies failed to find any positive effects of mindfulness on conscious control components in the process of practicing mindfulness (Malinowski et al., 2017; Norris et al., 2018). On the other hand, mindfulness was found to have less conscious control over task-independent stimuli (Slagter et al., 2007; Howells et al., 2012; Moore et al., 2012; Atchley et al., 2016). However, CCC can limit the occurrence of this automatic attention to some extent (Hofmann et al., 2009). The level of CCC can reflect an individual’s control ability to overcome immediate stimuli (Hofmann et al., 2009). Therefore, individuals with high levels of cognitive control capacity may have lower levels of trait mindfulness.

Furthermore, it’s important to note that in most studies, the cognitive tasks were performed immediately after the mindfulness intervention. In mindful meditation, the neural signature of attention could be detected very early, just a few minutes after the start of mindful intervention (Lakey et al., 2011). This rapid effect was found to be particularly pronounced for concentration (Chiesa et al., 2011). But would the effect last? If not, it can be argued that the effects reported after the long-term intervention may simply result from the participant’s most recent mindfulness training (Fjorback et al., 2011). Our study compared a stable level of cognitive ability with a stable level of mindfulness, with the intention of shedding light on the discrepancies between our findings and the hypotheses proposed regarding the positive association between cognitive control capacity (CCC) and trait mindfulness.

Inconsistent with our hypothesis, the positive relationship between trait mindfulness and sleep quality was not significant. This can be explained by the fact that mindfulness is complex and not always beneficial (Hafenbrack and Vohs, 2018; Britton, 2019). Indeed, a study highlighted a crucial turning point in meditation practices, where below a certain point the practices facilitate sleep and above which they tend to inhibit sleep (Britton et al., 2010). These findings shed light on the potential effects of meditation practices on sleep patterns, with implications for individuals seeking to optimize their sleep quality through mindfulness exercises. Low practice volume in subjects of Mindfulness-Based Cognitive Therapy increased sleep duration, but as practice volume approached 30 min per day, sleep duration and depth began to decrease and cortical arousal (awakenings and micro-arousals) started to increase. Long-term meditators were also found to have worse sleep than non-meditators, with cortical arousal linearly correlated with lifetime meditation practice volume (Ferrarelli et al., 2013). Additionally, studies comparing different types of practice of mindfulness found that body awareness exercises were to a lesser extent associated with unwanted effects caused by mindfulness, while attention exercises were more often related to unwanted effects (Cebolla et al., 2017).

Furthermore, we failed to find that CCC was significantly correlated with emotional distress, and the mediation effect of emotional distress proved to be insignificant. Perhaps this is because the association between cognitive control and emotions is more reflected in emotional regulation and may have little impact on emotional distress (Tice and Bratslavsky, 2000).

This study has several strengths. It used CCC as an independent variable to investigate its effect on sleep quality. Conceptually, the CCC can probe the upper limit of cognitive control of information processing, and the inclusion of trait mindfulness and emotional distress can help investigate the mechanisms underlying sleep quality and cognitive control. Methodologically, we adopted the MFT-M-R paradigm to measure CCC, which can more accurately reflect the level of individual cognitive control. From an educational perspective, the current findings may advance our understanding of the relationship between sleep and cognitive control in adolescents, offering insights into prospective intervention strategies. We identified trait mindfulness and emotional distress as two important mediators and depicted how they worked. Our main findings have implications for the development and refinement of interventions aimed at overcoming sleep problems in adolescents. Specifically, given the important role of self-control, interventions can incorporate techniques to enhance self-control. In addition, we should upgrade our understanding of the role of mindfulness. The practice of mindfulness may serve as a feasible technique for counterbalancing the influence of unconscious representations (Dehaene, 2018), compensating for the inherent instability of self-control in adolescents, improving self-regulation and potentially buffering the adverse effects of emotional distress on sleep health. Therefore, interventions such as school-based mindfulness practices and group counseling are interventions that can be focused on in future research.

Despite its findings, the present study has several limitations. First, the sample of participants was limited for they were recruited only from senior high school students in a Chinese middle school. Therefore, it may not be adequately representative of the adolescent population in general. It is recommended that future research endeavors aim to improve the diversity of participants’ backgrounds. This would help to ensure a more comprehensive understanding of the effects of mindfulness practices on various populations. Such an approach would enhance the generalizability and applicability of research findings, ultimately benefiting individuals from diverse cultural, social, and economic backgrounds. Secondly, due to the long duration (40 min) of the MFT-M-R experiment, this study collected valid data from only 149 participants. The limited sample size might have caused sampling error. Therefore, increasing the sample size to improve the reliability of the results is necessary. Last, we employed a two-wave longitudinal design. Such a design is very commonly used in organizational psychology (e.g., Burić et al., 2019; Muntz and Dormann, 2020; Spagnoli et al., 2021) and has advantages over the cross-sectional design. However, as some researchers suggest that the minimum number of waves in a longitudinal design should be three (e.g., Ployhart and Vandenberg, 2010), future research could include more waves of data collection when examining the relationships between CCC, trait mindfulness, emotional distress, and sleep quality, particularly if researchers are interested in exploring potential mediators. Besides, given the myriad definitions of mindfulness and different components of trait mindfulness, further research could investigate the nuances of trait mindfulness and how the varying components may individually affect sleep quality. This would allow for targeted clinical interventions to focus not only on practices of teaching mindfulness but also on finding which aspects of mindfulness are most useful in improving sleep quality. Future scholars could explore the possible mechanisms behind the association between different components of mindfulness and sleep quality, facilitating clinical interventions more effectively. Finally, this study yielded several results that were not consistent with the hypothesis, and the reasons for these results require further research.

## 5 Conclusion

This study contributes to our understanding of how cognitive control capacity impacts sleep quality. We found that CCC was indirectly associated with poor sleep quality in a sequential mediation first through trait mindfulness and then through emotional distress. The study provides implications for future exploration of the mechanism behind the relationship between cognitive control and sleep quality, as well as practical solutions for sleep problems, including clinical interventions.

## Data availability statement

The raw data supporting the conclusions of this article will be made available by the authors, without undue reservation.

## Ethics statement

The studies involving humans were approved by Ethics Committee of the School of Psychology, South China Normal University. The studies were conducted in accordance with the local legislation and institutional requirements. Written informed consent for participation in this study was provided by each participant’s legal guardian or next of kin.

## Author contributions

YW: Data curation, Formal analysis, Investigation, Methodology, Writing – original draft, Writing – review & editing. HL: Data curation, Writing – original draft, Writing – review & editing. XL: Writing – original draft, Writing – review & editing. BZ: Writing – original draft, Writing – review & editing. MH: Writing – original draft, Writing – review & editing. CC: Data curation, Formal analysis, Investigation, Methodology, Writing – original draft, Writing – review & editing.

## Footnotes

^1^ www.mathworks.cn/products/matlab.html

^2^ https://www.ibm.com/spss

## References

[ref1] Aivaliotis V. I. Lee Y. Zia J. Wassef W. Abramson M. Park W. (2017). Telephone-based mindfulness therapy intervention for patients with chronic pancreatitis. Dig. Dis. Sci. 62, 502–509. doi: 10.1007/s10620-016-4389-6, PMID: 27933469

[ref2] Atchley R. Klee D. Memmott T. Goodrich E. Wahbeh H. Oken B. (2016). Event-related potential correlates of mindfulness meditation competence. Neuroscience 320, 83–92. doi: 10.1016/j.neuroscience.2016.01.051, PMID: 26850995 PMC4777645

[ref3] Baer R. A. Smith G. T. Lykins E. Button D. Krietemeyer J. Sauer S.. (2008). Construct validity of the five facet mindfulness questionnaire in meditating and nonmeditating samples. Assessment 15, 329–342. doi: 10.1177/1073191107313003, PMID: 18310597

[ref4] Bahani M. Zhang Y. Guo Y. Haretebieke S. Wu D. Zhang L. (2024). Influencing factors of sleep quality in pregnant: a structural equation model approach. BMC Psychol 12:171. doi: 10.1186/s40359-024-01657-1, PMID: 38528622 PMC10964610

[ref5] Baumeister R. F. Bratslavsky E. Muraven M. Tice D. M. (1998). Ego depletion: is the active self a limited resource? J. Pers. Soc. Psychol. 74, 1252–1265. doi: 10.1037/0022-3514.74.5.1252, PMID: 9599441

[ref6] Baumeister R. F. Tice D. M. Vohs K. D. (2018). The strength model of self-regulation: conclusions from the second decade of willpower research. Perspect. Psychol. Sci. 13, 141–145. doi: 10.1177/1745691617716946, PMID: 29592652

[ref7] Baumeister R. F. Vohs K. D. Tice D. M. (2007). The strength model of self-control. Curr. Dir. Psychol. Sci. 16, 351–355. doi: 10.1111/j.1467-8721.2007.00534.x

[ref8] Bishop S. R. Lau M. Shapiro S. Carlson L. Anderson N. D. Carmody J.. (2004). Mindfulness: a proposed operational definition. Clin. Psychol. Sci. Pract. 11, 230–241. doi: 10.1093/clipsy.bph077

[ref9] Brisbon N. M. Lachman M. E. (2017). Dispositional mindfulness and memory problems: the role of perceived stress and sleep quality. Mindfulness 8, 379–386. doi: 10.1007/s12671-016-0607-8, PMID: 28344682 PMC5363402

[ref10] Britton W. B. (2019). Can mindfulness be too much of a good thing? The value of a middle way. Curr. Opin. Psychol. 28, 159–165. doi: 10.1016/j.copsyc.2018.12.011, PMID: 30708288 PMC6612475

[ref11] Britton W. B. Haynes P. L. Fridel K. W. Bootzin R. R. (2010). Polysomnographic and subjective profiles of sleep continuity before and after mindfulness-based cognitive therapy in partially remitted depression. Psychosom. Med. 72, 539–548. doi: 10.1097/PSY.0b013e3181dc1bad, PMID: 20467003

[ref12] Brown K. W. Ryan R. M. (2003). The benefits of being present: mindfulness and its role in psychological well-being. J. Pers. Soc. Psychol. 84, 822–848. doi: 10.1037/0022-3514.84.4.822 12703651

[ref13] Brown K. W. Weinstein N. Creswell J. D. (2012). Trait mindfulness modulates neuroendocrine and affective responses to social evaluative threat. Psychoneuroendocrinology 37, 2037–2041. doi: 10.1016/j.psyneuen.2012.04.003, PMID: 22626868 PMC5087919

[ref14] Burić I. Slišković A. Penezić Z. (2019). A two-wave panel study on teachers’ emotions and emotional-labour strategies. Stress. Health 35, 27–38. doi: 10.1002/smi.2836, PMID: 30194896

[ref15] Burke T. M. Scheer F. A. J. L. Ronda J. M. Czeisler C. A. Wright K. P. (2015). Sleep inertia, sleep homeostatic and circadian influences on higher-order cognitive functions. J. Sleep Res. 24, 364–371. doi: 10.1111/jsr.12291, PMID: 25773686 PMC5124508

[ref16] Buysse D. J. Reynolds C. F. Monk T. H. Berman S. R. Kupfer D. J. (1989). The Pittsburgh sleep quality index: a new instrument for psychiatric practice and research. Psychiatry Res. 28, 193–213. doi: 10.1016/0165-1781(89)90047-4 2748771

[ref17] Cebolla A. Demarzo M. Martins P. Soler J. Garcia-Campayo J. (2017). Unwanted effects: is there a negative side of meditation? A multicentre survey. PLoS ONE 12:e0183137. doi: 10.1371/journal.pone.0183137, PMID: 28873417 PMC5584749

[ref18] Chiesa A. Calati R. Serretti A. (2011). Does mindfulness training improve cognitive abilities? A systematic review of neuropsychological findings. Clin. Psychol. Rev. 31, 449–464. doi: 10.1016/j.cpr.2010.11.003, PMID: 21183265

[ref19] Conley S. L. Faleer H. E. Raza G. T. Bailey B. E. Wu K. D. (2018). The moderating effects of rumination facets on the relationship between mindfulness and distress reduction. Cogn. Ther. Res. 42, 436–446. doi: 10.1007/s10608-018-9896-7

[ref20] Dehaene S. (2018). The error-related negativity, self-monitoring, and consciousness. Perspect. Psychol. Sci. 13, 161–165. doi: 10.1177/1745691618754502, PMID: 29592636

[ref21] Drapeau A. Marchand A. Beaulieu-Prevost D. (2012). “Epidemiology of psychological distress” in Mental illnesses—understanding, prediction and control. ed. LAbate L. (InTech). Open. www.intechopen.com.

[ref22] Ebneabbasi A. Mahdipour M. Nejati V. Li M. Liebe T. Colic L.. (2021). Emotion processing and regulation in major depressive disorder: a 7T resting-state fMRI study. Hum. Brain Mapp. 42, 797–810. doi: 10.1002/hbm.25263, PMID: 33151031 PMC7814754

[ref23] Eisenlohr-Moul T. A. Peters J. R. Pond R. S. DeWall C. N. (2016). Both trait and state mindfulness predict lower aggressiveness via anger rumination: a multilevel mediation analysis. Mindfulness 7, 713–726. doi: 10.1007/s12671-016-0508-x, PMID: 27429667 PMC4943669

[ref24] Evans J. (2008). Dual-processing accounts of reasoning, judgment, and social cognition. Annu. Rev. Psychol. 59, 255–278. doi: 10.1146/annurev.psych.59.103006.093629, PMID: 18154502

[ref25] Evans L. Haeberlein K. Chang A. Handal P. (2020). An evaluation of the convergent validity of and preliminary cutoff scores for the DASS-21 total score as a measure of distress in adolescents. Curr. Psychol. 41, 4283–4290. doi: 10.1007/s12144-020-00937-4

[ref26] Fan J. (2014). An information theory account of cognitive control. Front. Hum. Neurosci. 8. doi: 10.3389/fnhum.2014.00680, PMID: 25228875 PMC4151034

[ref27] Fazio R. H. Towles-Schwen T. (1999). “The MODE model of attitude-behavior processes” in Dual-process theories in social psychology. eds. J. W. Sherman, B. Gawronski, & Y. Trope. (New York, NY: The Guilford Press), 97–116.

[ref28] Ferrarelli F. Smith R. Dentico D. Riedner B. A. Zennig C. Benca R. M.. (2013). Experienced mindfulness meditators exhibit higher parietal-occipital EEG gamma activity during NREM sleep. PLoS One 8:e73417. doi: 10.1371/journal.pone.0073417, PMID: 24015304 PMC3756031

[ref29] Fjorback L. O. Arendt M. Ørnbøl E. Fink P. Walach H. (2011). Mindfulness-based stress reduction and mindfulness-based cognitive therapy — a systematic review of randomized controlled trials. Acta Psychiatr. Scand. 124, 102–119. doi: 10.1111/j.1600-0447.2011.01704.x 21534932

[ref30] Frewen P. A. Evans E. M. Maraj N. Dozois D. J. A. Partridge K. (2008). Letting go: mindfulness and negative automatic thinking. Cogn. Ther. Res. 32, 758–774. doi: 10.1007/s10608-007-9142-1

[ref31] Gadie A. Shafto M. Leng Y. Kievit R. A. (2017). How are age-related differences in sleep quality associated with health outcomes? An epidemiological investigation in a UK cohort of 2406 adults. BMJ Open 7:e014920. doi: 10.1136/bmjopen-2016-014920, PMID: 28760786 PMC5642766

[ref32] Galla B. M. (2016). Within-person changes in mindfulness and self-compassion predict enhanced emotional well-being in healthy, but stressed adolescents. J. Adolesc. 49, 204–217. doi: 10.1016/j.adolescence.2016.03.016, PMID: 27107398

[ref33] Gilbert B. D. Christopher M. S. (2010). Mindfulness-based attention as a moderator of the relationship between depressive affect and negative cognitions. Cogn. Ther. Res. 34, 514–521. doi: 10.1007/s10608-009-9282-6

[ref34] Goldstein R. Z. Volkow N. D. (2011). Dysfunction of the prefrontal cortex in addiction: neuroimaging findings and clinical implications. Nat. Rev. Neurosci. 12, 652–669. doi: 10.1038/nrn3119, PMID: 22011681 PMC3462342

[ref35] Gong X. Xie X. Xu R. Luo Y. (2010). Psychometric properties of the Chinese versions of DASS-21 in Chinese college students. Chin. J. Clin. Psych. 18, 443–446. doi: 10.16128/j.cnki.1005-3611.2010.04.020

[ref36] Goschke T. Bolte A. (2014). Emotional modulation of control dilemmas: the role of positive affect, reward, and dopamine in cognitive stability and flexibility. Neuropsychologia 62, 403–423. doi: 10.1016/j.neuropsychologia.2014.07.015, PMID: 25068705

[ref37] Gradisar M. Kahn M. Micic G. Short M. Reynolds C. Orchard F.. (2022). Sleep’s role in the development and resolution of adolescent depression. Nat. Rev. Psychol. 1, 512–523. doi: 10.1038/s44159-022-00074-8, PMID: 35754789 PMC9208261

[ref38] Guller B. Yaylaci F. (2024). Eating and sleep problems, related factors, and effects on the mental health of the parents in children with autism spectrum disorder. Int. J. Develop. Disabil. 70, 406–415. doi: 10.1080/20473869.2022.2095689, PMID: 38699491 PMC11062261

[ref39] Hafenbrack A. C. Vohs K. D. (2018). Mindfulness meditation impairs task motivation but not performance. Organ. Behav. Hum. Decis. Process. 147, 1–15. doi: 10.1016/j.obhdp.2018.05.001

[ref40] Hall M. Buysse D. J. Nowell P. D. Nofzinger E. A. Houck P. Reynolds C. F.. (2000). Symptoms of stress and depression as correlates of sleep in primary insomnia. Psychosom. Med. 62, 227–230. doi: 10.1097/00006842-200003000-00014 10772402

[ref41] Hayes A. F. (2017). Introduction to mediation, moderation, and conditional process analysis. 2nd Edn. New York, NY: The Guilford Press.

[ref42] He X. Qiu B. Deng Y. Liu T. Chen Y. Zhang W. (2022). Adaptive assessment of the capacity of cognitive control. Q. J. Exp. Psychol. 75, 43–52. doi: 10.1177/17470218211030838 34165352

[ref43] He H. Xu P. Wu T. Chen Y. Wang J. Qiu Y.. (2019). Reduced capacity of cognitive control in older adults with mild cognitive impairment. JAD 71, 185–200. doi: 10.3233/JAD-181006 31356201

[ref44] Heatherton T. F. Wagner D. D. (2011). Cognitive neuroscience of self-regulation failure. Trends Cogn. Sci. 15, 132–139. doi: 10.1016/j.tics.2010.12.005, PMID: 21273114 PMC3062191

[ref45] Hofmann W. Baumeister R. F. Förster G. Vohs K. D. (2012). Everyday temptations: An experience sampling study of desire, conflict, and self-control. J. Pers. Soc. Psychol. 102, 1318–1335. doi: 10.1037/a0026545, PMID: 22149456

[ref46] Hofmann W. Friese M. Strack F. (2009). Impulse and self-control from a dual-systems perspective. Perspect. Psychol. Sci. 4, 162–176. doi: 10.1111/j.1745-6924.2009.01116.x, PMID: 26158943

[ref47] Hofmann S. G. Sawyer A. T. Witt A. A. Oh D. (2010). The effect of mindfulness-based therapy on anxiety and depression: a meta-analytic review. J. Consult. Clin. Psychol. 78, 169–183. doi: 10.1037/a0018555, PMID: 20350028 PMC2848393

[ref48] Hölzel B. K. Carmody J. Evans K. C. Hoge E. A. Dusek J. A. Morgan L.. (2010). Stress reduction correlates with structural changes in the amygdala. Soc. Cogn. Affect. Neurosci. 5, 11–17. doi: 10.1093/scan/nsp034, PMID: 19776221 PMC2840837

[ref49] Howells F. M. Ives-Deliperi V. L. Horn N. R. Stein D. J. (2012). Mindfulness based cognitive therapy improves frontal control in bipolar disorder: a pilot EEG study. BMC Psychiatry 12:15. doi: 10.1186/1471-244X-12-15, PMID: 22375965 PMC3305658

[ref50] Hsu S.-C. Wang S.-J. Liu C.-Y. Juang Y.-Y. Yang C.-H. Hung C.-I. (2009). The impact of anxiety and migraine on quality of sleep in patients with major depressive disorder. Compr. Psychiatry 50, 151–157. doi: 10.1016/j.comppsych.2008.07.002, PMID: 19216892

[ref51] Kabat-Zinn J. (1990). Full catastrophe living: Using the wisdom of your body and mind to face stress, pain and illness. New York, NY: Dell Publishing.

[ref52] Kabat-Zinn J. (1994). Wherever you go, there you are: Mindfulness meditation in everyday life. New York, NY: Hyperion.

[ref53] Khoury B. Sharma M. Rush S. E. Fournier C. (2015). Mindfulness-based stress reduction for healthy individuals: a meta-analysis. J. Psychosom. Res. 78, 519–528. doi: 10.1016/j.jpsychores.2015.03.009, PMID: 25818837

[ref54] Kiken L. G. Shook N. J. (2012). Mindfulness and emotional distress: the role of negatively biased cognition. Personal. Individ. Differ. 52, 329–333. doi: 10.1016/j.paid.2011.10.031

[ref55] Kiken L. G. Shook N. J. (2014). Does mindfulness attenuate thoughts emphasizing negativity, but not positivity? J. Res. Pers. 53, 22–30. doi: 10.1016/j.jrp.2014.08.002, PMID: 25284906 PMC4178287

[ref56] Kroese F. M. Evers C. Adriaanse M. A. de Ridder D. T. (2016). Bedtime procrastination: a self-regulation perspective on sleep insufficiency in the general population. J. Health Psychol. 21, 853–862. doi: 10.1177/1359105314540014, PMID: 24997168

[ref57] Lai H. Good M. (2005). Music improves sleep quality in older adults. J. Adv. Nurs. 49, 234–244. doi: 10.1111/j.1365-2648.2004.03281.x 15660547

[ref58] Lakey C. E. Berry D. R. Sellers E. W. (2011). Manipulating attention via mindfulness induction improves P300-based brain computer interface performance. J. Neural Eng. 8:025019. doi: 10.1088/1741-2560/8/2/025019, PMID: 21436516 PMC4429763

[ref59] Latif I. Hughes A. T. L. Bendall R. C. A. (2019). Positive and negative affect mediate the influences of a maladaptive emotion regulation strategy on sleep quality. Front. Psych. 10:628. doi: 10.3389/fpsyt.2019.00628, PMID: 31543841 PMC6730659

[ref60] Lawlor M. S. Schonert-Reichl K. A. Gadermann A. M. Zumbo B. D. (2014). A validation study of the mindful attention awareness scale adapted for children. Mindfulness 5, 730–741. doi: 10.1007/s12671-013-0228-4

[ref61] Li Y. (2017). The correlation between mindfulness attention awareness and anxiety in middle school students. Mental Health Education in Primary and Secondary. 25, 15–17.

[ref62] Liu Y. Chen C. Du H. Xue M. Zhu N. (2024). Impact of Baduanjin exercise combined with rational emotive behavior therapy on sleep and mood in patients with poststroke depression: a randomized controlled trial. Medicine 103:e38180. doi: 10.1097/MD.0000000000038180, PMID: 38728460 PMC11081619

[ref63] Liu Q. Niu G. Fan C. Zhou Z. (2017). Passive use of social network site and its relationships with self-esteem and self-concept clarity: a moderated mediation analysis. Acta Psychol. Sin. 49:60. doi: 10.3724/SP.J.1041.2017.00060

[ref64] Liu L. Su T. Peng J. Guo Y. Feng T. (2014). The cognitive and neural mechanism of the delay discounting: from the trait and state perspectives. Adv. Psychol. Sci. 22:1047. doi: 10.3724/SP.J.1042.2014.01047

[ref65] Liu X. Tang M. Hu L. Wang A. Wu Z. Gao C.. (1996). Reliability and validity of the Pittsburgh sleep quality index. Chin. J. Psychiatry, 29, 103–107.

[ref66] Liu H. Wu X. Deng Y. (2018). Mindfulness and depression of college students: the chain mediation of experience avoidance and verbose thinking. J. Clin. Psychol. 26, 565–569. doi: 10.16128/j.cnki.1005-3611.2018.03.031

[ref67] Luo Y. Du J. Wang J. Liu P. Shi Z. He Y.. (2024). Progressive muscle relaxation alleviates anxiety and improves sleep quality among healthcare practitioners in a mobile cabin hospital: a pre-post comparative study in China. Front. Psychol. 15:1337318. doi: 10.3389/fpsyg.2024.1337318, PMID: 38746917 PMC11091277

[ref68] Mackie M.-A. Fan J. (2016). Reduced efficiency and capacity of cognitive control in autism spectrum disorder: cognitive control in ASD. Autism Res. 9, 403–414. doi: 10.1002/aur.1517, PMID: 26171787 PMC4713391

[ref69] MacKinnon D. P. Krull J. L. Lockwood C. M. (2000). Equivalence of the mediation, confounding and suppression effect. Prev. Sci. 1, 173–181. doi: 10.1023/A:1026595011371, PMID: 11523746 PMC2819361

[ref70] Malinowski P. Moore A. W. Mead B. R. Gruber T. (2017). Mindful aging: the effects of regular brief mindfulness practice on electrophysiological markers of cognitive and affective processing in older adults. Mindfulness 8, 78–94. doi: 10.1007/s12671-015-0482-8, PMID: 28163795 PMC5241348

[ref71] Moore A. Gruber T. Derose J. Malinowski P. (2012). Regular, brief mindfulness meditation practice improves electrophysiological markers of attentional control. Front. Hum. Neurosci. 6:18. doi: 10.3389/fnhum.2012.00018, PMID: 22363278 PMC3277272

[ref72] Mueller C. Nenert R. Catiul C. Pilkington J. Szaflarski J. P. Amara A. W. (2024). Relationship between sleep, physical fitness, brain microstructure, and cognition in healthy older adults: a pilot study. Brain Res. 1839:149016. doi: 10.1016/j.brainres.2024.149016, PMID: 38768934

[ref73] Muntz J. Dormann C. (2020). Moderating effects of appreciation on relationships between illegitimate tasks and intrinsic motivation: a two-wave shortitudinal study. Eur. J. Work Organ. Psy. 29, 391–404. doi: 10.1080/1359432X.2019.1706489

[ref74] Norris C. J. Creem D. Hendler R. Kober H. (2018). Brief mindfulness meditation improves attention in novices: evidence from ERPs and moderation by neuroticism. Front. Hum. Neurosci. 12:315. doi: 10.3389/fnhum.2018.00315, PMID: 30127731 PMC6088366

[ref75] Ployhart R. E. Vandenberg R. J. (2010). Longitudinal research: the theory, design, and analysis of change. J. Manag. 36, 94–120. doi: 10.1177/0149206309352110

[ref76] Punamäki R. Wallenius M. Nygård C. Saarni L. Rimpelä A. (2007). Use of information and communication technology (ICT) and perceived health in adolescence: the role of sleeping habits and waking-time tiredness. J. Adolesc. 30, 569–585. doi: 10.1016/j.adolescence.2006.07.004, PMID: 16979753

[ref77] Pychyl T. A. Sirois F. (2016). “Procrastination, emotion regulation, and well-being” in Procrastination, health, and well-being. eds. Sirois, F. M., & Pychyl, T. A. (San Diego: Elsvier Academic Press), 163–188.

[ref78] Sarchiapone M. Mandelli L. Carli V. Iosue M. Wasserman C. Hadlaczky G.. (2014). Hours of sleep in adolescents and its association with anxiety, emotional concerns, and suicidal ideation. Sleep Med. 15, 248–254. doi: 10.1016/j.sleep.2013.11.780, PMID: 24424101

[ref79] Schantz B. L. Toner E. R. Brown M. L. Kaiser N. Chen A. Adhikari S.. (2024). Examining the relationship between emotion regulation, sleep quality, and anxiety disorder diagnosis. J. Mood Anxiety Disord. 8:100072. doi: 10.1016/j.xjmad.2024.100072 PMC11661812 39711805

[ref80] Seo J.-H. Kim J. H. Yang K. I. Hong S. B. (2017). Late use of electronic media and its association with sleep, depression, and suicidality among Korean adolescents. Sleep Med. 29, 76–80. doi: 10.1016/j.sleep.2016.06.022, PMID: 27887887

[ref81] Shen L. van Schie J. Ditchburn G. Brook L. Bei B. (2018). Positive and negative emotions: differential associations with sleep duration and quality in adolescents. J. Youth Adolescence 47, 2584–2595. doi: 10.1007/s10964-018-0899-1 30039509

[ref82] Sirois F. (2014). Procrastination and stress: exploring the role of self-compassion. Self Identity 13, 128–145. doi: 10.1080/15298868.2013.763404

[ref83] Sirois F. Pychyl T. (2013). Procrastination and the priority of short-term mood regulation: consequences for future self: procrastination, mood regulation and future self. Soc. Personal. Psychol. Compass 7, 115–127. doi: 10.1111/spc3.12011

[ref84] Slagter H. A. Lutz A. Greischar L. L. Francis A. D. Nieuwenhuis S. Davis J. M.. (2007). Mental training affects distribution of limited brain resources. PLoS Biol. 5:e138. doi: 10.1371/journal.pbio.0050138, PMID: 17488185 PMC1865565

[ref85] Songkham W. Deeluea J. Suksatit B. Chaiard J. (2019). Sleep quality among industrial workers: related factors and impact. JHR 33, 119–126. doi: 10.1108/JHR-08-2018-0072

[ref86] Souders M. Mason T. Valladares O. Bucan M. Levy S. Mandell D.. (2009). Sleep behaviors and sleep quality in children with autism Spectrum disorders. Sleep, 32, 1566–1587. doi: 10.1093/sleep/32.12.1556 20041592 PMC2786040

[ref87] Spagnoli P. Kovalchuk L. S. Aiello M. S. Rice K. G. (2021). The predictive role of perfectionism on heavy work investment: a two-waves cross-lagged panel study. Personal. Individ. Differ. 173:110632. doi: 10.1016/j.paid.2021.110632

[ref88] Teasdale J. D. Segal Z. Williams J. M. G. (1995). How does cognitive therapy prevent depressive relapse and why should attentional control (mindfulness) training help? Behav. Res. Ther. 33, 25–39. doi: 10.1016/0005-7967(94)E0011-7, PMID: 7872934

[ref89] Tice D. M. Bratslavsky E. (2000). Giving in to feel Good: the place of emotion regulation in the context of general self-control. Psychol. Inq. 11, 149–159. doi: 10.1207/S15327965PLI1103_03

[ref90] Vernon L. Modecki K. L. Barber B. L. (2018). Mobile phones in the bedroom: trajectories of sleep habits and subsequent adolescent psychosocial development. Child Dev. 89, 66–77. doi: 10.1111/cdev.12836, PMID: 28556891

[ref91] Vignaud P. Donde C. Sadki T. Poulet E. Brunelin J. (2018). Neural effects of mindfulness-based interventions on patients with major depressive disorder: a systematic review. Neurosci. Biobehav. Rev. 88, 98–105. doi: 10.1016/j.neubiorev.2018.03.004, PMID: 29548932

[ref92] Vohs K. D. (2006). Self-regulatory resources power the reflective system: evidence from five domains. J. Consum. Psychol. 16, 217–223. doi: 10.1207/s15327663jcp1603_3

[ref93] Waters L. (2016). The relationship between child stress, child mindfulness and parent mindfulness. PSYCH 7, 40–51. doi: 10.4236/psych.2016.71006

[ref94] Way B. M. Creswell J. D. Eisenberger N. I. Lieberman M. D. (2010). Dispositional mindfulness and depressive symptomatology: correlations with limbic and self-referential neural activity during rest. Emotion 10, 12–24. doi: 10.1037/a0018312, PMID: 20141298 PMC2868367

[ref95] Wen Z. Ye B. (2014). Analyses of mediating effects: the development of methods and models. Adv. Psychol. Sci. 22:731. doi: 10.3724/SP.J.1042.2014.00731

[ref96] Williams P. G. Cribbet M. R. Rau H. K. Gunn H. E. Czajkowski L. A. (2013). The effects of poor sleep on cognitive, affective, and physiological responses to a laboratory stressor. Ann. Behav. Med. 46, 40–51. doi: 10.1007/s12160-013-9482-x, PMID: 23504562

[ref97] Wu Q. Chang C.-F. Xi S. Huang I.-W. Liu Z. Juan C.-H.. (2015). A critical role of temporoparietal junction in the integration of top-down and bottom-up attentional control: interaction of top-down and bottom-up attention. Hum. Brain Mapp. 36, 4317–4333. doi: 10.1002/hbm.22919, PMID: 26308973 PMC4619130

[ref98] Wu T. Chen C. Spagna A. Wu X. Mackie M. Russell-Giller S.. (2020). The functional anatomy of cognitive control: a domain-general brain network for uncertainty processing. J. Comp. Neurol. 528, 1265–1292. doi: 10.1002/cne.24804, PMID: 31674015

[ref99] Wu T. Dufford A. J. Mackie M.-A. Egan L. J. Fan J. (2016). The capacity of cognitive control estimated from a perceptual decision making task. Sci. Rep. 6:34025. doi: 10.1038/srep34025, PMID: 27659950 PMC5034293

[ref100] Xiao C. Mou C. Zhou Z. (2019). Effect of mindfulness meditation training on anxiety, depression and sleep quality in perimenopausal women. J. Southern Medical Univ. 39, 998–1002. doi: 10.12122/j.issn.1673-4254.2019.08.19, PMID: 31511223 PMC6765589

[ref101] Xie M. Zhang Y. Wang W. Chen H. Lin D. (2024). Associations between multiple dimensions of sleep and mood during early adolescence: a longitudinal daily diary study. J. Youth Adolescence. doi: 10.1007/s10964-024-02007-5, PMID: 38753280

[ref102] Xu W. Fu G. An Y. Yuan G. Ding X. Zhou Y. (2018). Mindfulness, posttraumatic stress symptoms, depression, and social functioning impairment in Chinese adolescents following a tornado: mediation of posttraumatic cognitive change. Psychiatry Res. 259, 345–349. doi: 10.1016/j.psychres.2017.09.088, PMID: 29120841

[ref103] Yosiaki S. Ogawa M. Kawada T. Suzuki S. Duki M. Z. (1998). Afternoon exercise improves the quality of night sleep: a case study observed by EEG and self-rating scale. J. Occup. Health 40, 37–43. doi: 10.1539/joh.40.37

[ref104] Zeidan F. Johnson S. K. Diamond B. J. David Z. Goolkasian P. (2010). Mindfulness meditation improves cognition: evidence of brief mental training. Conscious. Cogn. 19, 597–605. doi: 10.1016/j.concog.2010.03.014, PMID: 20363650

[ref105] Zhai X. Zeng J. Eshak E. S. Zhang Y. Yang M. Di L.. (2021). The influencing factors of sleep quality among Chinese junior and senior high school adolescents during the COVID-19 pandemic. J. Trop. Pediatr. 67:fmab069. doi: 10.1093/tropej/fmab069, PMID: 34329443 PMC8385850

